# Resistance-associated substitutions and response to treatment in a chronic hepatitis C virus infected-patient: an unusual virological response case report

**DOI:** 10.1186/s12879-021-06080-0

**Published:** 2021-04-26

**Authors:** Fabián Aldunate, Natalia Echeverría, Daniela Chiodi, Pablo López, Adriana Sánchez-Cicerón, Martín Soñora, Juan Cristina, Gonzalo Moratorio, Nelia Hernández, Pilar Moreno

**Affiliations:** 1 Laboratorio de Virología Molecular, Centro de Investigaciones Nucleares, Facultad de Ciencias, Universidad de la República, Mataojo 2055, ZIP: 11400 Montevideo, Uruguay; 2 Laboratorio de Evolución Experimental de Virus, Institut Pasteur de Montevideo, Mataojo 2020, ZIP: 11400 Montevideo, Uruguay; 3 Clínica de Gastroenterología, Hospital de Clínicas, Facultad de Medicina, Universidad de la República, Av. Italia s/n, ZIP: 11600 Montevideo, Uruguay; 4 Laboratorio de Patología Clínica, Hospital de Clínicas, Facultad de Medicina, Universidad de la República, Av. Italia s/n, ZIP: 11600 Montevideo, Uruguay; 5 Laboratorio de Simulaciones Biomoleculares, Institut Pasteur de Montevideo, Mataojo 2020, ZIP: 11400 Montevideo, Uruguay

**Keywords:** DAA therapy, Hepatitis C virus, RASs minority variants, Relapse, Case report

## Abstract

**Background:**

Direct-Acting agents (DAAs) target and inhibit essential viral replication proteins. They have revolutionized the treatment of Hepatitis C virus (HCV) infection reaching high levels of sustained virologic response. However, the detection of basal resistance-associated substitutions (RASs) to DAAs in *naïve* patients could be important in predicting the treatment outcome in some patients exhibiting failures to DAA-based therapies. Therefore, the aim of this work was to evaluate the presence of RASs as minority variants within intra-host viral populations, and assess their relationship to response to therapy on a multiple times relapser patient infected chronically with HCV.

**Case presentation:**

A male HCV infected-patient with a genotype 1a strain was evaluated. He had previously not responded to dual therapy (pegylated interferon-α plus ribavirin) and was going to start a direct-acting agent-based therapy (DAAs). He showed no significant liver fibrosis (F0). Viral RNA was extracted from serum samples taken prior and after therapy with DAAs (sofosbubir/ledipasvir/ribavirin). NS5A and NS5B genomic regions were PCR-amplified and the amplicons were sequenced using Sanger and next-generation sequencing (NGS) approaches. RASs were searched in *in-silico* translated sequences for all DAAs available and their frequencies were determined for those detected by NGS technology. Sanger sequencing did not reveal the presence of RASs in the consensus sequence neither before nor after the DAA treatment. However, several RASs were found at low frequencies, both before as well as after DAA treatment. RASs found as minority variants (particularly substitutions in position 93 within NS5A region) seem to have increased their frequency after DAA pressure. Nevertheless, these RASs did not become dominant and the patient still relapsed, despite perfect adherence to treatment and having no other complications beyond the infection (no significant fibrosis, no drug abuse).

**Conclusions:**

This report shows that some patients might relapse after a DAA-based therapy even when RASs (pre- and post-treatment) are detected in very low frequencies (< 1%) within intra-host viral populations. Increased awareness of this association may improve detection and guide towards a personalized HCV treatment, directly improving the outcome in hard-to-treat patients.

**Supplementary Information:**

The online version contains supplementary material available at 10.1186/s12879-021-06080-0.

## Background

Hepatitis C virus (HCV) infects around 71 million people worldwide [1], and is a leading cause of chronic liver diseases including cirrhosis and hepatocellular carcinoma. Nevertheless, many chronically infected patients remain asymptomatic over the years and only become aware of their infection once they develop cirrhosis [2].

HCV is an RNA virus belonging to the Hepacivirus genus within the *Flaviviridae* family and like many other RNA viruses, HCV exhibits a high mutation rate. This explains why HCV circulates, as a cloud of genetically related variants, termed quasiespecies. This is one of the main obstacles to prevent and treat this infection. Nowadays no effective prophylactic vaccine against HCV exists. However, the development of new Direct-Acting agents (DAAs), which target and inhibit essential viral replication proteins (NS3, NS5A, NS5B), has revolutionized the treatment of this infection reaching high levels of sustained virologic response (SVR).

Although these DAAs seem to be very effective in curing most of the infected patients, several obstacles remain for their broad implementation including limited access to therapy, and the difficulty in treating patients with comorbidities and/or those with advanced cirrhosis or HCC [3].

Concomitantly with the development of these drugs, and taking into account the evolutionary rate of this virus, resistant variants have emerged. These variants are defined by the presence of one or more specific resistance-associated substitutions (RASs). The detection of basal RASs to DAAs in *naïve* patients could be important in predicting the treatment outcome in some patients exhibiting failures to DAA-based therapies (5–10%) [4, 5]. Additionally, some reports show that the presence of RASs as minority or low frequency variants in the viral population could influence the final prognosis of the patient [6].

Here, we report a case of a chronic hepatitis C infected patient followed and treated unsuccessfully through the years with no other complication beyond the infection with this virus. These results not only illustrate the difficulties that clinicians go through when treating these patients, but also suggest a possible molecular contributing factor to the treatment failure.

## Case presentation

### Patient characteristics

A Uruguayan 58-year-old Caucasian male patient was diagnosed with a chronic hepatitis C infection on May 2012 (genotype 1a and a viral load of 6.3 logs). HCV infection was detected by real time PCR using Abbott Real Time HCV kit (Abbott Molecular Inc., Des Plaines, USA) and viral genotype was confirmed by amplification of the Okamoto region of the NS5B polymerase gene and subsequent phylogenetic analyses. Both tests for hepatitis B surface antigen and human immunodeficiency virus were negative. The patient’s medical history suggested no hepatic risk of liver diseases and no history of alcohol or drug abuse. Both the patient and his family denied having a history of other risk factors for HCV infection. Liver biopsy did not show any significant fibrosis (F0).

### Treatment

**Fig. 1:**
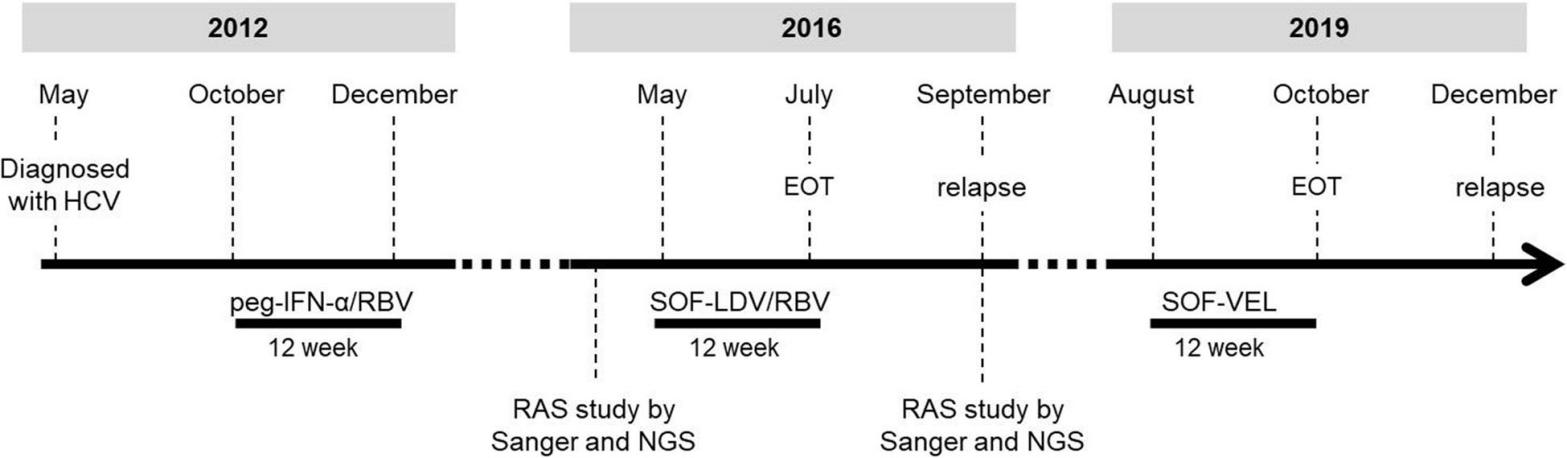
Patient treatment timeline. Initial diagnosis, periods of therapies and responses to treatment are indicated on top of the time line, whereas type of treatment and samples used for RAS analyses in this study are indicated below

On October 2012 the patient started the classic pharmacologic treatment with pegylated interferon-alpha (peg-IFN-α) (180 μg/week) in combination with ribavirin (RBV) (1000 mg/day) (Fig. 1). Treatment was well tolerated. On weeks 4 and 12 of treatment, the patient was evaluated by PCR and still showed detectable viral RNA levels (6.7 logs viral load at week 12). Therefore, the patient was considered as non-responder (NR) which led to the interruption of the classic treatment. Since the suspension of the dual therapy and up to December 2015 the patient received no other medical treatment for HCV and there was no evidence of fibrosis progression (measured by elastography).

On May 2016, when HCV viral load was 6.8 logs, the patient started a 12-week DAA-based treatment with 400 mg/day of sofosbuvir (SOF) and 90 mg/day of ledipasvir (LDV) plus RBV (1000 mg/day) with self-reported good adherence to it and no concomitant medication. Serum HCV RNA became negative during therapy (week 4), and it remained so at end of treatment (EOT_SOF/LDV/RBV_) as well. However, 12 weeks after EOT_SOF/LDV/RBV_, the serum revealed the presence of viral RNA with a viral load of 7.1 logs. Therefore, the patient was considered as a relapser to this first DAA-therapy. On August 2019, the patient started a second 12-week DAA-based treatment, in this case, with 400 mg/day of SOF and 100 mg/day of velpatasvir (VEL) (Fig. 1). Fibrosis had progressed (F3, measured by elastography). Once again, HCV RNA levels became undetectable during and at EOT_SOF/VEL_, but unfortunately, the patient suffered a new relapse (7.0 logs viral load 12 weeks after EOT_SOF/VEL_).

### RASs analyses by sanger and next generation sequencing (NGS)

In order to explain the treatment failures observed, serum samples from the patient taken before (April 2016) and after treatment with SOF/LDV + RBV (3 months after EOT_SOF/LDV/RBV_) were analyzed. Since the antivirals used were directed to inhibit viral proteins NS5A (LDV) and NS5B (SOF), viral RNA was extracted and both genomic regions were PCR-amplified (using a high-fidelity polymerase). The unlikely possibility of reinfection was discarded by phylogenetic analyses of the strains under study (Supplementary Material). Then, with the aim of identifying RASs that might be responsible for the relapse, the PCR products from NS5A and NS5B genomic regions were sequenced using the Sanger methodology. Next, we pursued a deeper analysis of the samples by Next Generation Sequencing (NGS) in an effort to identify viral resistant variants present in minority frequencies (0.1–1.0%) within the viral population. To this end, the amplified PCR products were used for NGS library construction using the TruSeq Nano DNA LT Library Prep Kit (Illumina, San Diego, CA, USA) and a paired-end sequencing was run on a Mi-Seq instrument (Illumina). RASs were searched in *in-silico* translated sequences for all direct acting agents (DAAs) available to date by means of the on-line tool Geno2pheno [hcv] [7] and their frequencies were determined for those detected from NGS technology with the software UnifiedGenotyper (Genome Analysis Toolkit) [8] using the MPileUp algorithm [9].

**Fig. 2:**
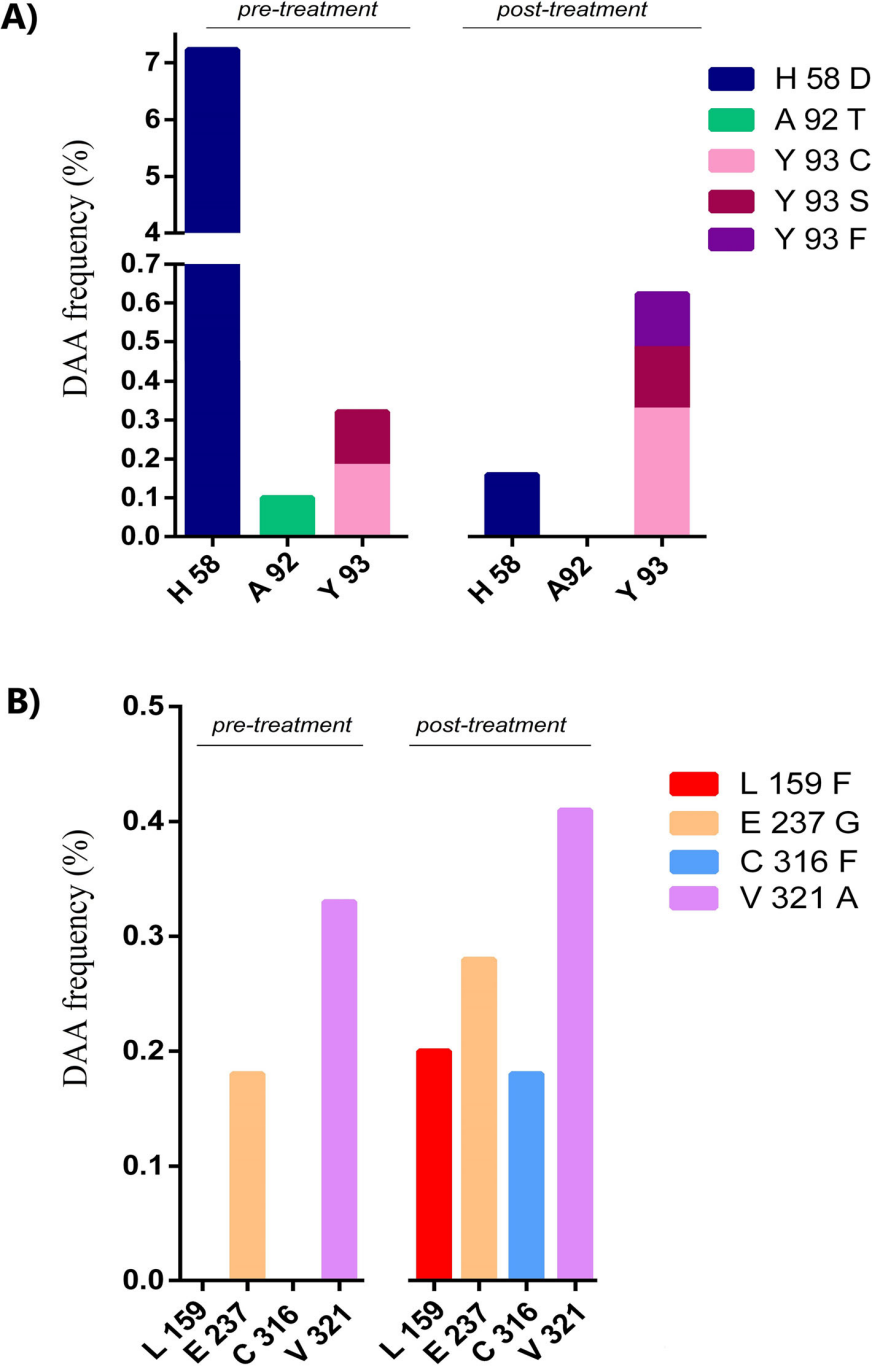
RASs found pre- and post-treatment with LDV/SOF + RBV detected as low frequency variants by NGS. **2a** RASs conferring resistance to LDV detected within NS5A genomic region. **2b** RASs conferring resistance to SOF detected within NS5B genomic region

The analyses of Sanger sequencing results did not reveal the presence of RASs in the consensus sequences neither before nor after the DAA treatment with SOF/LDV. On the other hand, several RASs were found by NGS approach both before as well as after DAA treatment. Some of them were found as minority variants (0.1–1.0%) and a few also as low frequency variants (1.0–15.0%), both in NS5A and in NS5B genomic regions (see Fig. 2a and b).

Within NS5A region, three amino acidic (aa) positions exhibited RASs to LDV (Fig. 2a) before treatment started (H58D, A92T and Y93C/S). The changes that have been reported to have an important role in conferring LDV resistance were changes in position 93 (Paolucci et al., 2013). LDV seems to have exerted pressure on the distribution of the viral population since RASs in this position increased their frequencies (Y93C/S from 0.33% to Y93C/S/F - 0.65%), and even a new change in this position was detected (Y93F in a frequency of 0,14%).

Contrary to what was expected, H58D frequency drastically diminished (from 7,23% to 0,16%) and A92T, only marginally present before treatment (0,10%) was no longer found after treatment.

Within the NS5B region, two RASs to SOF were found as minority variants (E237G and V321A) both before as well as after treatment. In both substitutions, a slight increase in their frequencies was observed after treatment (Fig. 2b). It is also worth noting the emergence of RASs as minority variants in two different positions after the drug pressure (L159F – 0,20% and C316F – 0,18%) (Fig. 2b).

## Discussion and conclusions

In this study we report the presence of RASs to HCV NS5A and NS5B inhibitors as minority variants (less than 1%) in a relapser patient, both before and after treatment with DAAs. It is of note that we did not detect any basal RASs by Sanger sequencing.

Given that the patient did not present any comorbidities, no concomitant medications or significant fibrosis before therapy started and that he presented perfect adherence to it, he was expected to respond to this IFN-free regimen (SOF/LDV + RBV) and to reach an SVR. However, the patient relapsed 12 weeks after EOT.

The presence of clinically relevant RASs has been associated to lower SVR rates when found pre-treatment (basal RASs). It is commonly accepted that they must be present as high frequency variants (> 15%) within the viral populations to affect treatment outcome in a significant way [4, 5, 10]. Still, controversy exists about whether detection of mutations below that threshold, may have an impact in treatment outcome [11]. A fact that is clear though, is that many relapser patients exhibit clinically relevant RASs (detected by Sanger sequencing) at the time of virologic failure [12–14]. However, there are only a few reports showing that those RASs are selected from pre-existing low frequency variants which emerged as dominant mutations after DAA pressure [6, 15], highlighting the importance of monitoring RASs which go undetected through Sanger sequencing.

Importantly, the RASs found here were previously undetected by Sanger sequencing but they were already present at low frequencies. In addition, they have increased their frequency after DAA pressure (See Fig. 2). In this case, the different RASs found did not become dominant after treatment and the patient still relapsed. The fact that the post-treatment sample was taken 12 weeks after the EOT raises the question of the persistence of the RASs when the selective pressure was removed for almost 3 months. Especially if certain RASs could affect viral fitness, they might go back to low frequencies even if during treatment, they became high frequency variants. However, Paolucci et al, (2017) [4] described the presence of low frequency RASs in 60% of patients subjected to DAA therapy who achieved SVR. Their work suggests that these variants are not always subsequently selected for and might not influence therapy outcome. Nevertheless, the RASs detected in the relapser patient here could have played a role in the virologic failure given that no other risk factor was found to explain it.

Even though we did not analyze viral diversity after the second relapse to DAA-therapy (SOF/VEL), it should be taken into account that at this point the patient had already progressed to an advanced fibrosis state (F3), which led to an infection more difficult to treat. Additionally, some of the RASs detected after SOF/LDV/RBV EOT had increased their frequencies, and it is already documented that many NS5A RASs persist for long periods in the infected individual [16]. Since the RASs here detected also confer resistance to VEL, it might also be possible for them to have influenced the second DAA therapy if they were still present when the treatment started.

Our study addresses one possible contributing factor to an unusual therapeutic response with no clear association to any other clinical aspect. We cannot discard that other factors might have also contributed to the final outcome of the patient. Despite the existence of only a few reports linking single-nucleotide-polymorphisms (SNPs) near *IFN-λ3* gene (formerly known as *IL28B*) with response to IFN-free regimens [17, 18], the patient presented in this report harbored unfavorable genotypes both in rs12979860 (TT) and rs8099917 (GG). Furthermore, the presence of highly represented amino acid substitutions (not RASs) with potential predictive value of treatment failure [19], such as Q309R in NS5B, found as majority variant both pre and post SOF/LDV treatment, might also jointly account for the observed outcome. In addition, this work seeks to stress the contribution of NGS in clinical centers, in particular for those hard-to-treat patients that have relapsed at least once to a DAA-based therapy. To this aim, we recommend storing plasma samples before and 12 weeks after each DAA treatment in case a patient relapses, in order to have samples in hand if needed for NGS studies. Nowadays, NGS is becoming a more commonly used and affordable tool that might have an impact in better therapeutic decisions and potentially improving clinical outcomes. Unfortunately, a limitation of this work is that minority variants were not taken into account for the choice of treatment. This is mainly explained by the fact that currently, the only treatment financed by Uruguayan health insurance is the use of SOF plus daclatasvir. Any other optimal DAA treatment would need to be specifically requested and its financing could be denied.

This report demonstrates that some patients might relapse after a DAA-based therapy even when RASs (pre- and post-treatment) are detected in very low frequencies (< 1%). This fact highlights the importance of using NGS approaches to better understand treatment outcomes and tailor future therapy regimens that might be better suited to some DAA-experienced patients with no other well-documented risk factors that might account for a treatment failure. Increased awareness of this association may improve detection and guide towards a personalized HCV treatment, directly improving the outcome in hard-to-treat patients.

## Supplementary Information

**Additional file 1:.**

## Data Availability

The NGS fastq files generated and analysed during the current study are available in the SRA database and are accesible with the following link: https://www.ncbi.nlm.nih.gov/sra/PRJNA720615. The NS5A and NS5B consensus sequences obtained by Sanger sequencing have been submitted to GenBank database under accession numbers MW893638 to MW893641.

## Abbreviations

DAAs: Direct-Acting agents
EOT: End of Treatment
HCV: Hepatitis C Virus
LDV: Ledipasvir
NGS: Next Generation Sequencing
NR: Non-responder
peg-IFN-α: Pegylated Interferon alpha
RASs: Resistance-associated substitutions
RBV: Ribavirin
SOF: Sofosbuvir
SVR: Sustained Virological Response
VEL: Velpatasvir
