# Supplementary material for: Resistance-associated substitutions and response to treatment in a chronic hepatitis C virus infected-patient: an unusual virological response case report
Source: BMC Infect Dis. 2021 Apr 26;21:387. doi: 10.1186/s12879-021-06080-0 (PMC8077789; doi:10.1186/s12879-021-06080-0)
Supplement: Supplementary file 1 — Additional file 1:. [file 12879_2021_6080_MOESM1_ESM.pdf]

Supplementary Material – Aldunate et al 2021.

Phylogenetic analyses of the Case Report strains.

To study the possibility of reinfection in this patient we carry out a phylogenetic analysis of the strains detected before and after SOF/LDV treatment. First we included 15 Uruguayan 1a HCV strains with other 59 HCV strains isolated elsewhere, representing all HCV genotypes and main subtypes. Then we compared our patient strains only with strains belonging only to genotype 1. The HCV strains for the reinfection phylogenetic tests can be found in Aldunate et al. 2018, “Pretreatment Hepatitis C Virus NS5A/NS5B Resistance-Associated Substitutions in Genotype 1 Uruguayan Infected Patients”, Disease Markers. This phylogenetic study was made using the GTR + G + I model (General time reversible + gamma + invariant sites), maximum likelihood phylogenetic trees were constructed for NS5B using the MEGA 5.0 software (Tamura et al. 2011).

We analyzed the 361 nucleotides corresponding to the Okamoto region (positions 8265 to 8625, relative to strain H77 NC_004102) within the NS5B region. As a measure of the robustness of each node, we employed the bootstrapping method (200 pseudoreplicates).

The maximum likelihood phylogenetic tree (Supplementary figure 1) shows that the two strains corresponding to the pre- and post- SOF/LDV (shown in red) are highly related. Therefore, these results and the low probability of reinfection supports that both sequences correspond to the same variant that infected the patient.

A)

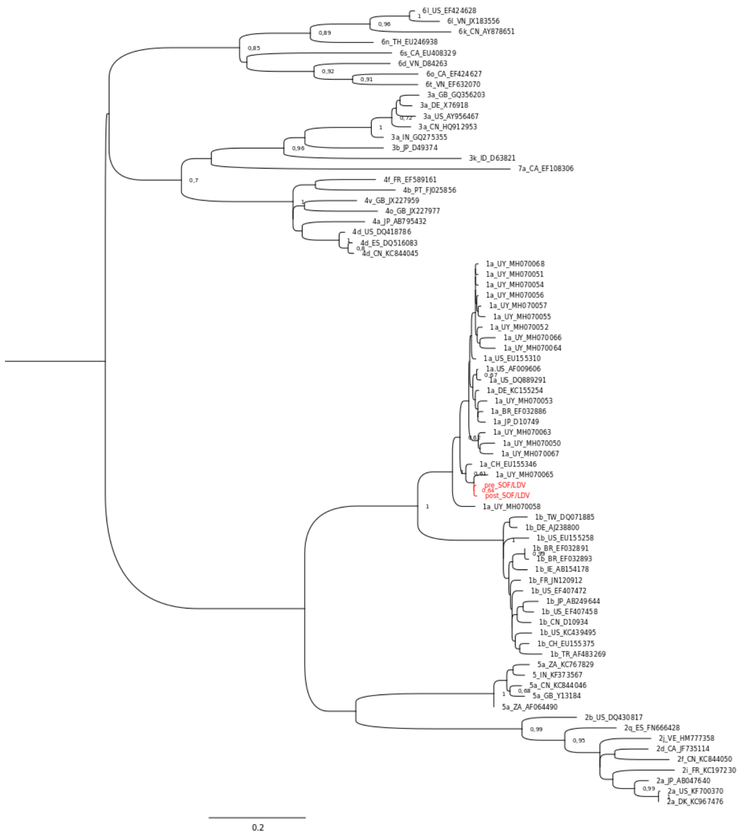

B)

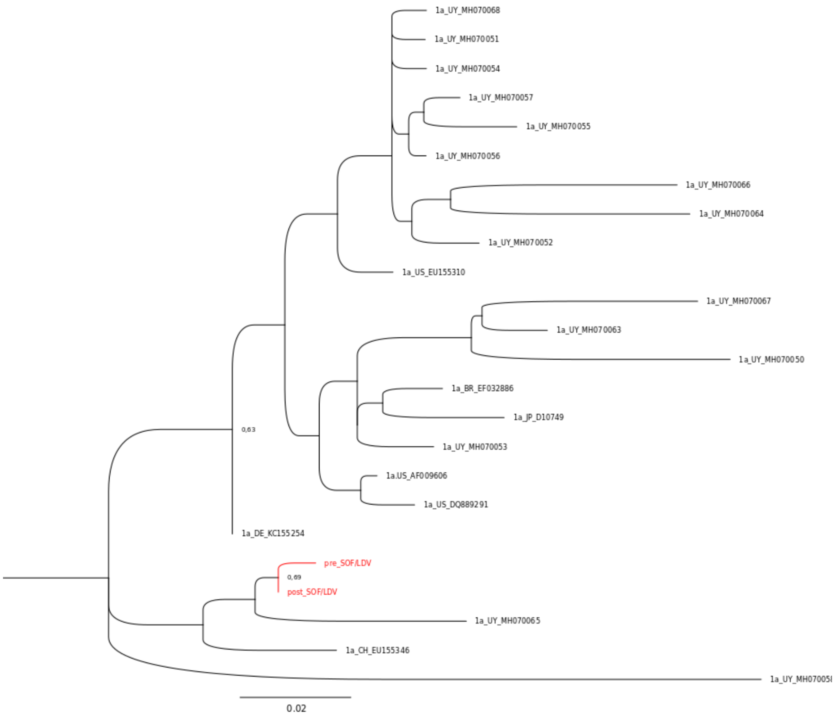

Supplementary figure 1. Maximum-likelihood phylogenetic tree analysis of the NS5B region (361 nucleotides) of HCV strains detected before and after SOF/LDV treatment. Reference strains in the tree are shown by genotype_country_accession number. Numbers at the branches indicate bootstrap values. The bar at the bottom of the tree denotes distance. The two strains corresponding to the pre- and post-SOF/LDV are shown in red. Maximum-likelihood phylogenetic tree including (A) all HCV genotypes and main subtypes and (B) including only 1a strain.
